# Error-prone DNA polymerase and oxidative stress increase the incidences of A to G mutations in tumors

**DOI:** 10.18632/oncotarget.13293

**Published:** 2016-11-11

**Authors:** Jiannan Lin, Tieliu Shi

**Affiliations:** ^1^ The Center for Bioinformatics and Computational Biology, Shanghai Key Laboratory of Regulatory Biology, The Institute of Biomedical Sciences and School of Life Sciences, East China Normal University, Shanghai 200241, China

**Keywords:** A to G mutation, mutational mechanism, error-prone DNA polymerase, oxidative stress, RAS mutation

## Abstract

Mutational processes for A→G mutations in tumors are not well understood. To uncover the mutational mechanisms, we analyzed molecular profiles of more than 9,000 tumor samples from The Cancer Genome Atlas (TCGA). The present study found that error-prone DNA polymerases were involved in stomach tumors with high fraction of A→G mutations. High levels of apoptosis in kidney cancers and high levels of energy metabolism in thyroid cancers increased A→G mutation rate, which was associated with high oxidative stress. We also found that the frequencies of RAS gene mutations were increased in thyroid cancers with high level of energy metabolism because of high-frequency A→G mutations.

## INTRODUCTION

Somatic mutations are essential for cancer formation and progression [1]. Exposure to exogenous mutagens can cause somatic mutations in cancers. For example, exposure to cigarette smoke causes a high proportion of C→A transversions in lung cancer [2–4]. Large numbers of C→T transitions at YpC (Y=C/T) dinucleotides in melanoma are attributed to ultraviolet light-induced cyclobutane pyrimidine dimers [5, 6]. Endogenous mutagens can also cause somatic mutations in cancers. Endogenous methyltransferases can methylate cytosines at CpG dinucleotides. Spontaneous deamination of the 5-methylcytosine base produces thymine and causes C-to-T mutation [7]. Activation-induced cytidine deaminases (AID) convert cytosine bases to uracil, which has been implicated in carcinogenesis [8]. Recent studies indicated that several homologous APOBEC cytidine deaminases induced C→T transitions in multiple tumor types [9, 10]. AIDs prefer adenine or guanine immediately 5′ to the target cytosine and APOBECs prefer thymine base 5′ to the target cytosine [11–13].

Fewer studies have focused on A→G (T→C) transitions in cancers. However, A→G (T→C) transitions may also be involved in the development of cancers. Predominance of A→G (T→C) transitions was observed in the genome of hepatitis C virus (HCV) positive hepatocellular carcinoma [14]. HCV-induced error-prone DNA polymerases may contribute to the high fraction of A→G (T→C) transitions [15]. High fraction of A→G (T→C) transitions was also reported in kidney cancer [7], but the mutational mechanism is unclear.

To investigate the mutational mechanism for high fraction of A→G (T→C) transitions in tumors, we performed a pan-cancer analysis on TCGA data. The result revealed that high fractions of A→G (T→C) transitions in stomach, kidney and thyroid cancers were closely associated with error-prone DNA polymerases or oxidative DNA damage.

## RESULTS

### A→G mutations in tumors

**Figure 1:**
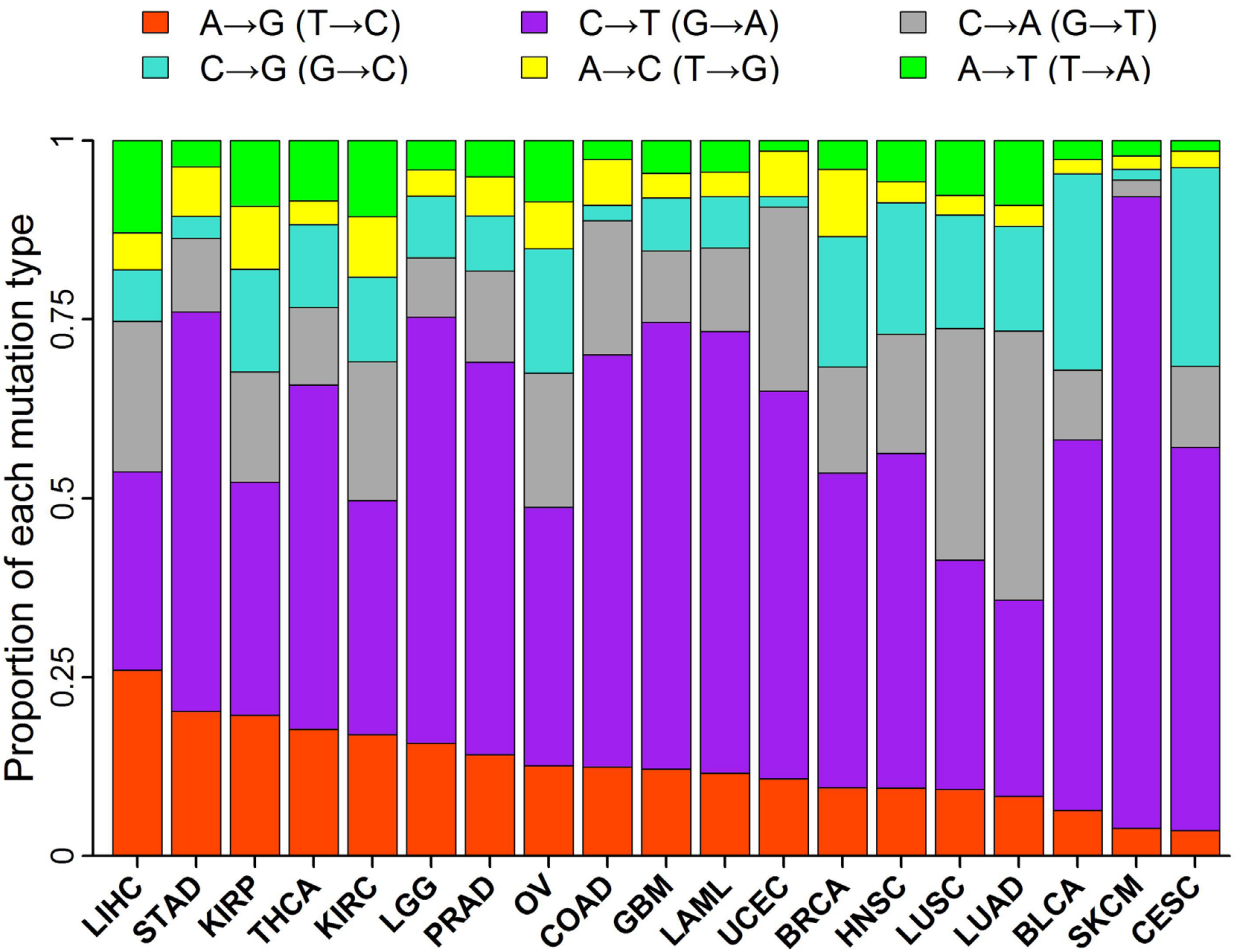
Proportion of each mutation type in 19 tumor types Stacked bar chart summarized the proportions of the six types of base-substitution mutations for each tumor type.

The fraction of A→G (T→C) mutations ranges from a low of 3.6% in cervical cancers (CESC) to a high of 26% in liver cancers (LIHC) (Figure 1). Low fractions of A→G mutations in cervical (CESC) and bladder (BLCA) cancers are largely attributed to high frequency of APOBEC-mediated C→T transitions [9, 10]. Low fractions of A→G mutations in skin cancers (SKCM) and lung cancers (LUSC and LUAD) are probably due to cyclobutane pyrimidine dimers induced by ultraviolet in skin and frequent C→A transversions induced by tobacco in lung [2, 3, 5, 16]. The tumor types with high fraction of A→G mutations included liver (LICH), stomach (STAD), kidney (KIRP and KIRC) and thyroid (THCA) cancers.

### Function enrichment for the genes associated with A→G mutation rate

We conducted function enrichment analyses on the top 100 genes having significant association between gene expression and A→G mutation rate. Function of translation was associated with A→G mutation rate in liver cancer (Supplementary Figure S1A). Our function enrichment analyses also associated cell cycle with A→G mutation rate in stomach cancers (Supplementary Figure S1B) and associated apoptosis with A→G mutation rate in renal clear cell carcinoma (Supplementary Figure S1D). In thyroid cancers, energy metabolism and mitochondrion organization were significantly correlated with A→G mutation rate (Supplementary Figure S1E).

### The high fraction of A→G mutations in stomach cancers is associated with error-prone DNA polymerases

**Figure 2:**
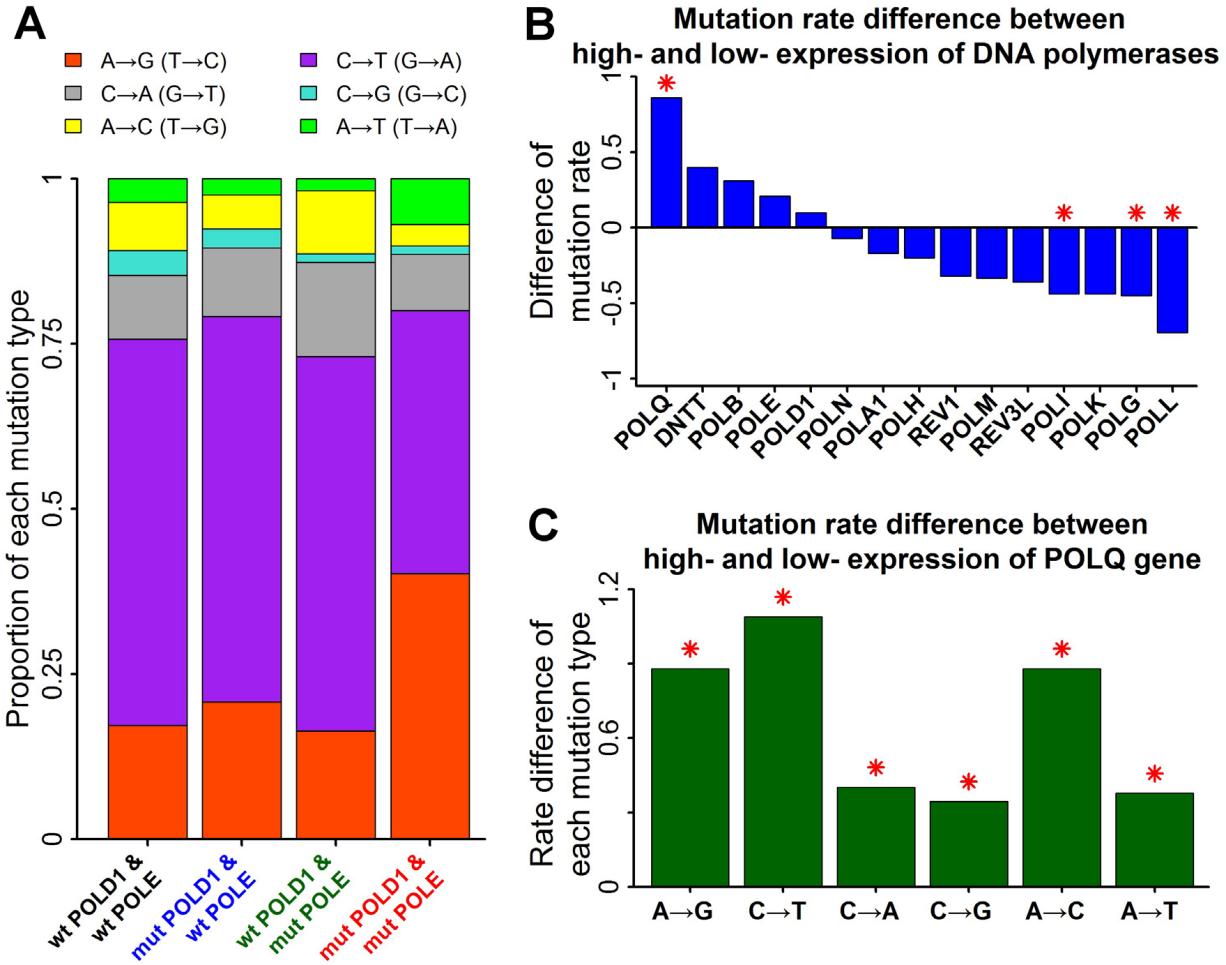
Mutation rate associated with error-prone DNA polymerases in stomach cancers **A**. Proportion of each mutation type associated with mutations on replicative DNA polymerases. Stacked bar chart summarized the proportions of the six types of base-substitution mutations for stomach cancers with replicative DNA polymerase mutations. “wt” represented wide-type tumors and “mut” represented mutant tumors. **B**. Mutation rate difference between high- and low- expression tumors of DNA polymerases. Stomach cancers with mutant POLD1 or mutant POLE were excluded for the subsequent analyses. Positive value represented higher mutation rate in high-expression tumors of DNA polymerases than low-expression tumors. Mutation rate was defined as the number of mutations per megabase. Red asterisks indicated significant difference of mutation rate (P<0.01 by Mann-Whitney U test). **C**. Mutation rate difference for all the six types of base-substitution mutations between high- and low- expression tumors of POLQ gene.

The function associated with A→G mutation rate in stomach cancers was cell cycle, implying the potential involvement of error-prone DNA polymerases. The fraction of A→G mutations was increased in stomach cancers with concurrent mutations on POLD1 and POLE genes (Figure 2A). Three replicative DNA polymerases have been identified in eukaryotes: DNA Polε (catalytic subunit: POLE) and Polδ (catalytic subunit: POLD1) synthesize the leading and lagging strands after priming by Pol α (catalytic subunit: POLA1) [17]. Concurrent defects of POLD1 and POLE may increase the chance of the involvement of error-prone DNA polymerases in DNA replication. Few alternation of A→G mutation frequency in cancers with only POLD1 or only POLE mutations can be explained by the strong function complementarity between POLD1 and POLE, reducing the influence of error-prone DNA polymerases.

In order to identify the DNA polymerase involved in the mutation induction, stomach cancers were divided into low-expression and high-expression groups according to gene expression levels of all the fifteen DNA polymerases in human [17]. Only the gene expression of POLQ (catalytic subunit of Polθ) was significantly associated with mutation induction in stomach cancers (Figure 2B). Polθ is a proofreading-deficient DNA polymerase involved in translesion DNA synthesis (TLS), which has a much lower fidelity than replicative DNA polymerases. Consistent with it, the mutation rates of the six types of base substitutions were all significantly increased in stomach cancers with high-expression POLQ (Figure 2C). High rate of A→G mutations led to an increase of the fraction of A→G mutations in stomach cancers with high-expression POLQ (Supplementary Figure S2).

### High-level apoptosis increases A→G mutation rate in kidney cancers

**Figure 3:**
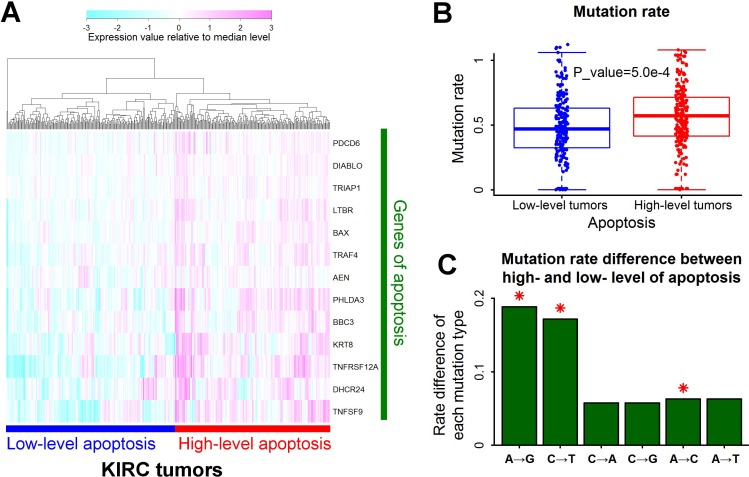
A→G mutation rate associated with apoptosis in renal clear cell carcinoma **A**. Tumors with different levels of apoptosis. Samples of renal clear cell carcinoma (KIRC) were clustered into two groups according to the expression level of genes responsible for apoptosis. The expression value was normalized by dividing with median level for each gene. **B**. Higher mutation rates in tumors with high-level apoptosis. Each data point represents one tumor sample. Mutation rate was defined as the number of mutations per megabase. The P value for mutation rate difference was estimated by Mann-Whitney U test. **C**. Mutation rate difference for all the six types of base substitutions between tumors with high- and low- level apoptosis. Positive value represented higher mutation rate in tumors with high-level apoptosis than low-level apoptosis. Red asterisks indicated significant difference of mutation rate (P<0.01 by Mann-Whitney U test).

According to the expression profile of 13 associated genes of apoptosis, tumors of renal clear cell carcinoma can be divided into two groups (Figure 3A). The mutation rate was much higher in tumors with high-level apoptosis than tumors with low-level apoptosis (Figure 3B). The elevation of A→G mutation rate was the highest in all the six types of base substitutions (Figure 3C). By contrast, no function was significantly associated with A→G mutation rate in renal papillary cell carcinoma (KIRP) (Supplementary Figure S1C), implying the different gene regulation between renal papillary cell carcinoma and renal clear cell carcinoma.

### High-level energy metabolism increases A→G mutation rate in thyroid cancers

**Figure 4:**
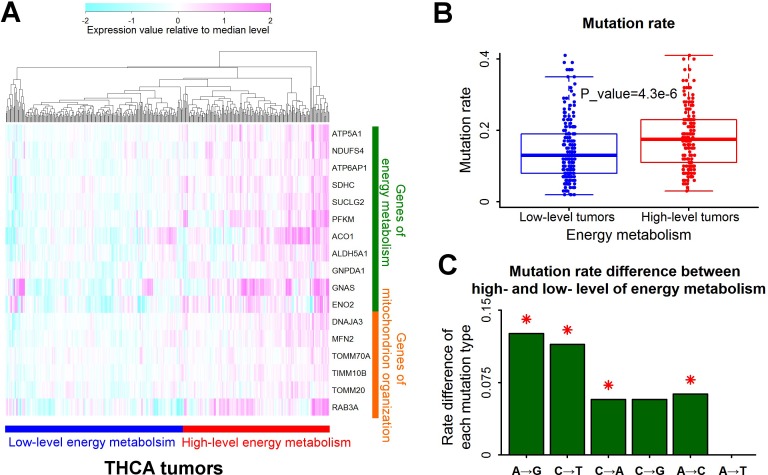
A→G mutation rate associated with energy metabolism in thyroid cancers **A**. Tumors with different levels of energy metabolism. Samples of thyroid cancers (THCA) were clustered into two groups according to the expression level of genes responsible for energy metabolism. The expression value was normalized by dividing with median level for each gene. **B**. Higher mutation rates in tumors with high-level energy metabolism. Each data point represents one tumor sample. Mutation rate was defined as the number of mutations per megabase. The P value for mutation rate difference was estimated by Mann-Whitney U test. **C**. Mutation rate difference for all the six types of base substitutions between tumors with high- and low- level energy metabolism. Positive value represented higher mutation rate in tumors with high-level energy metabolism than low-level energy metabolism. Red asterisks indicated significant difference of mutation rate (P<0.01 by Mann-Whitney U test).

Mitochondrion organization is an important biological process of energy metabolism. According to the expression profile of 11 associated genes of energy metabolism and 6 associated genes of mitochondrion organization, thyroid cancers were divided into two groups: low-level and high-level tumors of energy metabolism (Figure 4A). The mutation rate of tumors with high-level energy metabolism was significantly higher than tumors with low-level energy metabolism (Figure 4B). Like renal clear cell carcinoma, the elevation of A→G mutation rate by high-level energy metabolism was also the highest in all the six types of base substitutions in thyroid cancers (Figure 4C).

### A→G mutations prefer single-stranded DNA in kidney and thyroid cancers

Transcription not only is a key control point for gene expression but also affects DNA mutation rate. Transcription-coupled repair (TCR) recognizes the stalled RNA-polymerases on DNA lesions and initiates DNA repair on the transcribed strand [18, 19], reducing mutation rate of transcribed regions. On the other hand, transcribed regions are exposed to mutagens as single-strand DNA during synthesis of RNA transcripts and more prone to damage [20]. High levels of transcription are associated with increased mutation rates, which is termed transcription-associated mutation (TAM) [21]. Thus net impact of transcription on mutation rate can be used to measure the mutagenic strength of mutagens relative to TCR.

C→T mutation rate was detected in high-expressed genes for most tumor types (Supplementary Figure S3A), indicating that the effect of TCR overwhelms that of TAM. Only bladder cancers (BLCA) showed a weak elevation of C→T mutation rate in high-expressed genes. The elevation may be attributed to APOBEC mutagenesis. High-level APOBEC mutagenesis has been reported in bladder cancers, with a stringent signature TCN→TTN for C→T mutations [9, 10]. Therefore, we divided C→T mutations into two groups TCN→TTN and VCN→VTN (V base: all but T base) according to adjacent bases. The mutation rate of TCN→TTN was increased in high-expression genes of bladder cancers and cervical cancers (CESC: another tumor type enriched with APOBEC mutations) (Supplementary Figure S3B and S3C). Transcription produces single-stranded DNA, which is the ideal substrate of APOBEC enzymes [22]. In contrast, the mutation rate of VCN→VTN was decreased in high-expression genes, which can be attributed to TCR.

**Figure 5:**
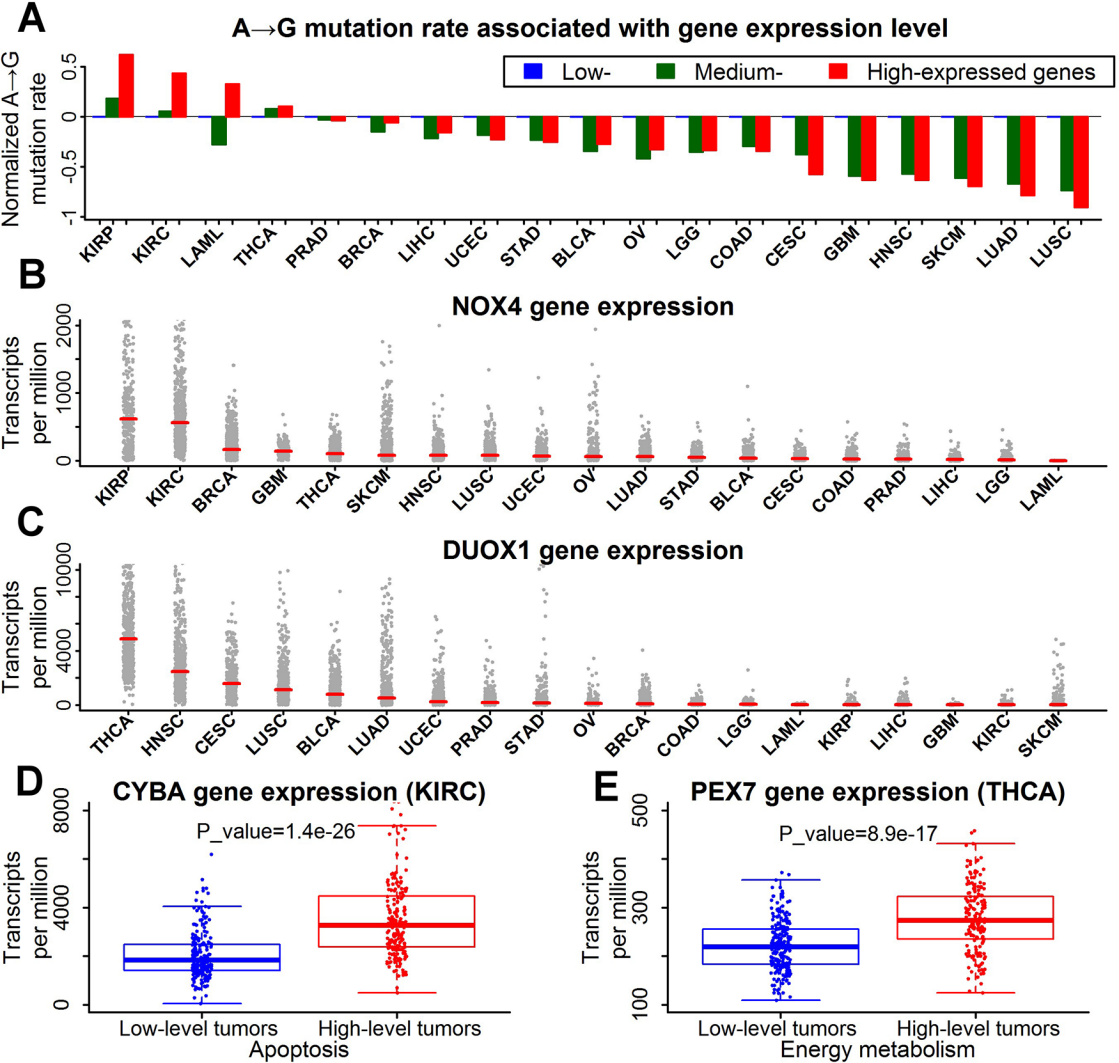
High fraction of A→G mutations was associated with oxidative stress in kidney and thyroid cancers **A**. Higher A→G mutation rates in high-expressed genes of kidney and thyroid cancers. Genes were categorized into three equal-size groups (low-, medium- and high-expressed) based on the rank of expression levels for each tumor type. The A→G mutation rates of each gene group were represented by the median value. Finally, the median rates of all gene groups were all normalized by dividing with the median rate of low-expressed genes. **B**. Expression levels of NOX4 gene across 19 tumor types. Each data point represents one tumor sample. Red horizontal lines indicated median fraction of each dataset. **C**. Expression levels of DUOX1 gene across 19 tumor types. **D**. Higher expression of CYBA gene in renal clear cell carcinoma with high-level apoptosis. Each data point represents one tumor sample. The P value for expression difference was estimated by Mann-Whitney U test. **E**. Higher expression of PEX7 gene in thyroid cancers with high-level energy metabolism.

Decreased A→G mutation rate was detected in high-expressed genes for most tumor types. However, as tumors with high fraction of A→G mutations, kidney cancers (KIRC and KIRP) and thyroid cancers (THCA) showed an elevation of A→G mutation rate in high-expressed genes (Figure 5A). It suggested that A→G mutations in kidney and thyroid cancers may be induced by mutagens that prefer single-stranded DNA.

An elevation of A→G mutation rate was also observed in high-expressed genes for acute myeloid leukemia (LAML) (Figure 5A). However, the elevation was only found in nonsynonymous substitution rate, rather than in synonymous substitution rate (Supplementary Figure S4A). The elevation of A→G mutation rate observed in acute myeloid leukemia may be derived from the positive selection of high-expressed genes. Unlike acute myeloid leukemia, synonymous and nonsynonymous substitution rate of other tumor types showed similar distributions for various gene expression levels (Supplementary Figure S4B-S4E).

### The high fraction of A→G mutations is associated with oxidative stress in kidney and thyroid cancers

Significant genes associated with A→G mutation rate were enriched in the cellar component of mitochondria for kidney (KIRC) and thyroid (THCA) cancers (Supplementary Figure S5A and S5B), implying the same mechanism for high fraction of A→G mutations. The most likely mutagen associated with mitochondria was oxidative mutagens. The mitochondrial respiratory chain is the major source of reactive oxygen species (ROS), which are constantly produced in the processes of apoptosis and energy metabolism [23–25]. ROS can cause various types of oxidative damage to DNA bases. 8-oxo-7,8 dihydroguanine (8-oxoG) is the most well-known DNA lesion of oxidative damage, resulting in G→A transition [26]. 8-oxoA is another common DNA lesion of oxidative damage, resulting in A→G transition [27]. Similar frequencies of 8-oxoA and 8-oxoG were observed in mammalian DNA [28, 29].

NADPH oxidases are the “professional” ROS producers in mammalian cells, which catalyze the transfer of electrons from NADPH to molecular oxygen [30]. The family of NADPH oxidases is composed of NOX1, NOX2, NOX3, NOX4, NOX5, DUOX1 and DUOX2 [31]. NOX1, NOX2, NOX3 and NOX4 are CYBA-dependent NADPH oxidases. Notably, NOX4 was predominantly expressed in kidney cancers (KIRC and KIRP) (Figure 5B and Supplementary Figure S6A). DUOX1 and DUOX2, formerly known as thyroid oxidases, were specifically expressed in thyroid cancers (THCA) (Figure 5C). The high expression of NADPH oxidases indicated high level of oxidative stress in kidney and thyroid cancers.

Gene expression of NOX4, DUOX1 and DUOX2 was not increased in kidney and thyroid cancers with high-level apoptosis and energy metabolism (data not shown), implying some other genes acting as the indicators of oxidative stress. The expression of gene CYBA, a critical component of NOX4 NADPH oxidase complex, was significantly increased by apoptosis in kidney cancers (KIRC) (Figure 5D). In thyroid cancers, the expression of peroxisomal-biogenesis genes PEX7, PEX11B and PEX19 was significantly increased by energy metabolism (Figure 5E and Supplementary Figure S6B-S6C). High level of peroxisomal biogenesis is associated with high level of oxidative stress [32].

High fraction of A→G mutations was associated with apoptosis in KIRC, but not in KIRP. Stronger depletion of mitochondria was observed in KIRC than KIRP [33], implying higher level of apoptosis in KIRC. But in KIRP, significant elevation of A→G mutation rate was observed in tumors with high expression of CYBA gene (Supplementary Figure S7), suggesting the association between oxidative stress and A→G mutations in KIRP.

### The high frequency of RAS mutations in thyroid cancers is attributed to A→G mutations induced by energy metabolism

**Figure 6:**
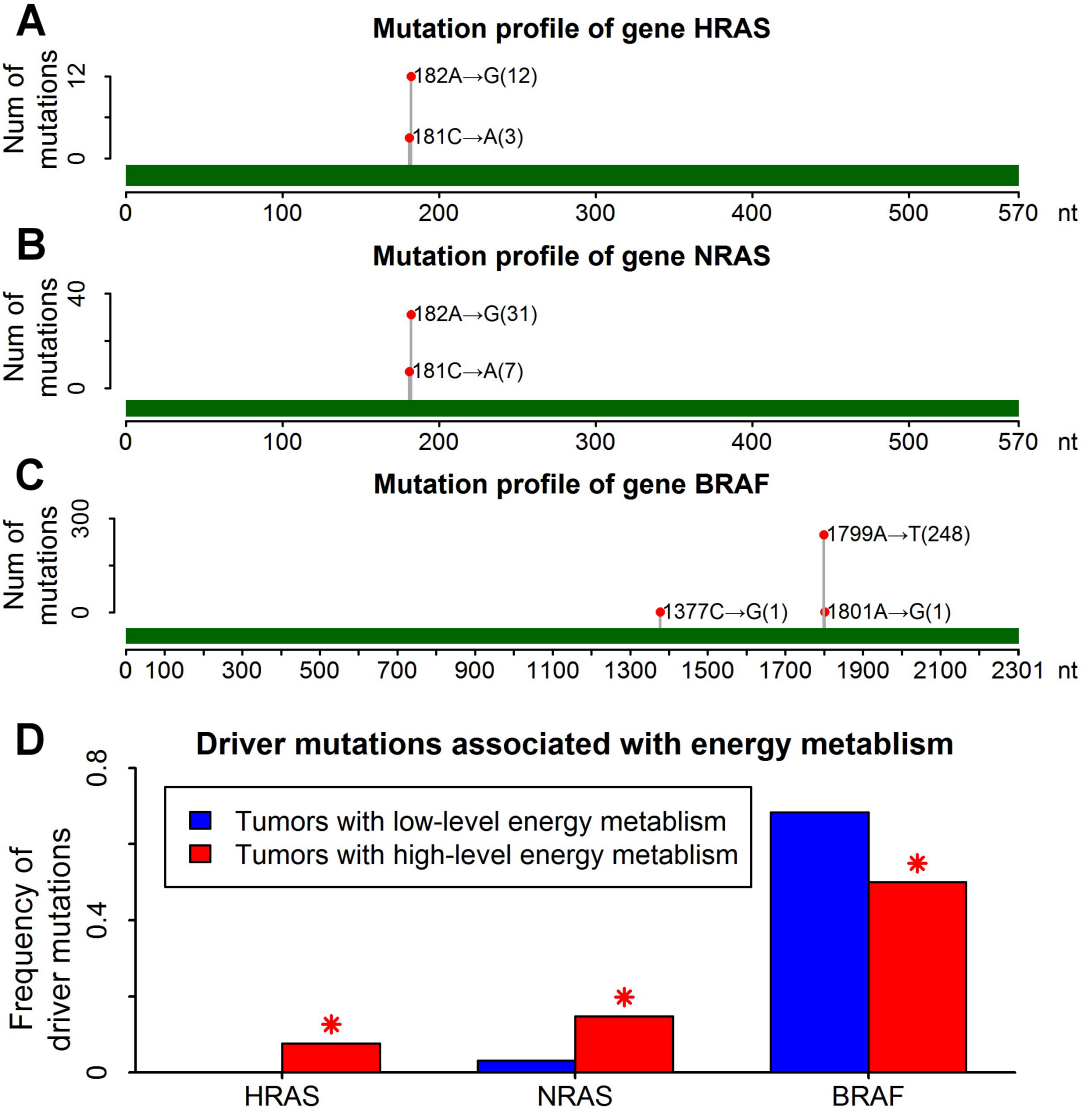
Higher frequency of RAS mutations in thyroid cancers with high-level energy metabolism **A**. Mutation profile of gene HRAS. Green bar showed the range of coding region. One red point indicated one recurrent mutation, following by the mutation position and mutation type. The number in parenthesis indicated the number of mutations. **B**. Mutation profile of gene NRAS. **C**. Mutation profile of gene BRAF. **D**. Driver mutations associated with energy metabolism in thyroid cancers. Blue and red bars indicated the mutation frequency of driver genes in tumors with low-level and high-level energy metabolism respectively. Red asterisks indicated significant difference of mutation frequency (P<0.01 by Fisher's exact test).

RAS (HRAS and NRAS) mutations are the second most common genetic alterations in thyroid cancers, which were mutually exclusive with BRAF mutations (the most common mutations in thyroid cancers) [34]. RAS-mutated thyroid cancers are aggressive tumors with a poor prognosis [35]. 81% of RAS-mutated thyroid cancers have a recurrent mutation of A→G mutation at nucleotide 182 of coding sequences (Figure 6A and 6B). A→G mutation rate was significantly increased in thyroid cancers with high-level energy metabolism (Figure 4C). Thus higher frequency of RAS (HRAS and NRAS) mutations was observed in thyroid cancers with high-level energy metabolism because of high-frequency A→G mutations (Figure 6D).

The vast majority of BRAF mutations in thyroid cancers were A→T mutations at nucleotide 1799 of coding sequences (Figure 6C). The frequency of BRAF mutations was significantly reduced in tumors with high-level energy metabolism (Figure 6D), which should result from the mutual exclusiveness between BRAF and RAS mutations. Excluding all the RAS-mutated tumors, similar frequencies of BRAF mutations were observed between tumors with low-level and high-level energy metabolism (Supplementary Figure S8).

## DISCUSSION

Oxidative DNA damage is very common in human cells. Oxidative mutagens can cause DNA damage which might increase the risk of cancer [36]. This study found that high-level oxidative stress may increase the frequency of RAS mutations in thyroid cancers with high-level energy metabolism. BRAF mutations were enriched in thyroid cancers with low-level energy metabolism because of the mutual exclusiveness between BRAF and RAS mutations. This finding may explain the lower level of iodine metabolism observed in mutant-BRAF thyroid cancers [37]. Iodine metabolism was highly correlated with energy metabolism (Supplementary Figure S9A-S9C).

HCV-induced error-prone DNA polymerases were reported to be involved in the induction of A→G (T→C) mutations of liver cancer [14]. But our function enrichment analyses did not found any association between DNA polymerases and A→G mutation rate in liver cancers, which may be the result of few liver cancers in TCGA affected by hepatitis C virus. Only one single liver cancer in TCGA expressed hepatitis C virus but at low levels [38]. Instead, we found significant associations between function of translation and A→G mutation rate in liver cancer. The connection between A→G mutations and function of translation in liver cancer was unclear.

This study found the association of A→G mutations with error-prone DNA polymerase and oxidative stress by bioinformatic approaches, which needs experimental validation in future. The association can be confirmed by the mutation profiles of human cell lines with reduced activity of error-prone DNA polymerase or oxidative stress. Future studies can examine the POLQ knockdown in stomach cancer cell lines with concurrent defects of POLD1 and POLE.

## MATERIALS AND METHODS

### TCGA datasets

Somatic mutations and expression profile were retrieved from The Cancer Genome Atlas (TCGA) on February 4, 2015. Tumor types with less than 100 individuals were excluded from the subsequent analyses to reduce the influence of small sample number. Point mutations were extracted from MAF files from the TCGA database. Mutation rate of each tumor was estimated for the six types of base substitutions. Mutant genes were defined as genes with non-silent somatic mutations. The expression level of each gene was extracted from the RSEM RNA-Seq data of TCGA.

### Function enrichment analysis

For each tumor type, Spearman correlation and associated P-value were estimated between expression level of each gene and A→G mutation rate in tumors. We filtered out the genes with P-values greater than 0.01. Then the top 100 genes with the positive correlation were defined as the significantly associated genes. Function enrichment analysis was performed on the significantly associated genes with DAVID tools [39].

### Mutation rate associated with gene expression level

Genes were categorized into equal-size groups based on the rank of expression levels for each tumor type. The median expression value was used to represent the expression level of each gene group. Median mutation rates of six types of base substitutions were estimated for each gene group.

### Synonymous and nonsynonymous substitution rate

Point mutations in protein-coding regions can be divided into synonymous (silent) and non-synonymous (amino acid-altering) mutations. The numbers of synonymous and non-synonymous sites of each gene were estimated using YN model implemented in KaKs_Calculator tools [40, 41]. Synonymous substitution rate was calculated as the number of synonymous substitutions per synonymous site [42, 43]. And non-synonymous substitution rate was calculated as the number of non-synonymous substitutions per non-synonymous site.

## SUPPLEMENTARY MATERIALS FIGURES


