# Supplementary material for: Error-prone DNA polymerase and oxidative stress increase the incidences of A to G mutations in tumors
Source: Oncotarget. 2016 Nov 11;8(28):45154–63. doi: 10.18632/oncotarget.13293 (PMC5542174; doi:10.18632/oncotarget.13293)
Supplement: Supplementary file 1 [file oncotarget-08-45154-s001.pdf]

# Error-prone DNA polymerase and oxidative stress increase the incidences of A to G mutations in tumors

## SUPPLEMENTARY FIGURES

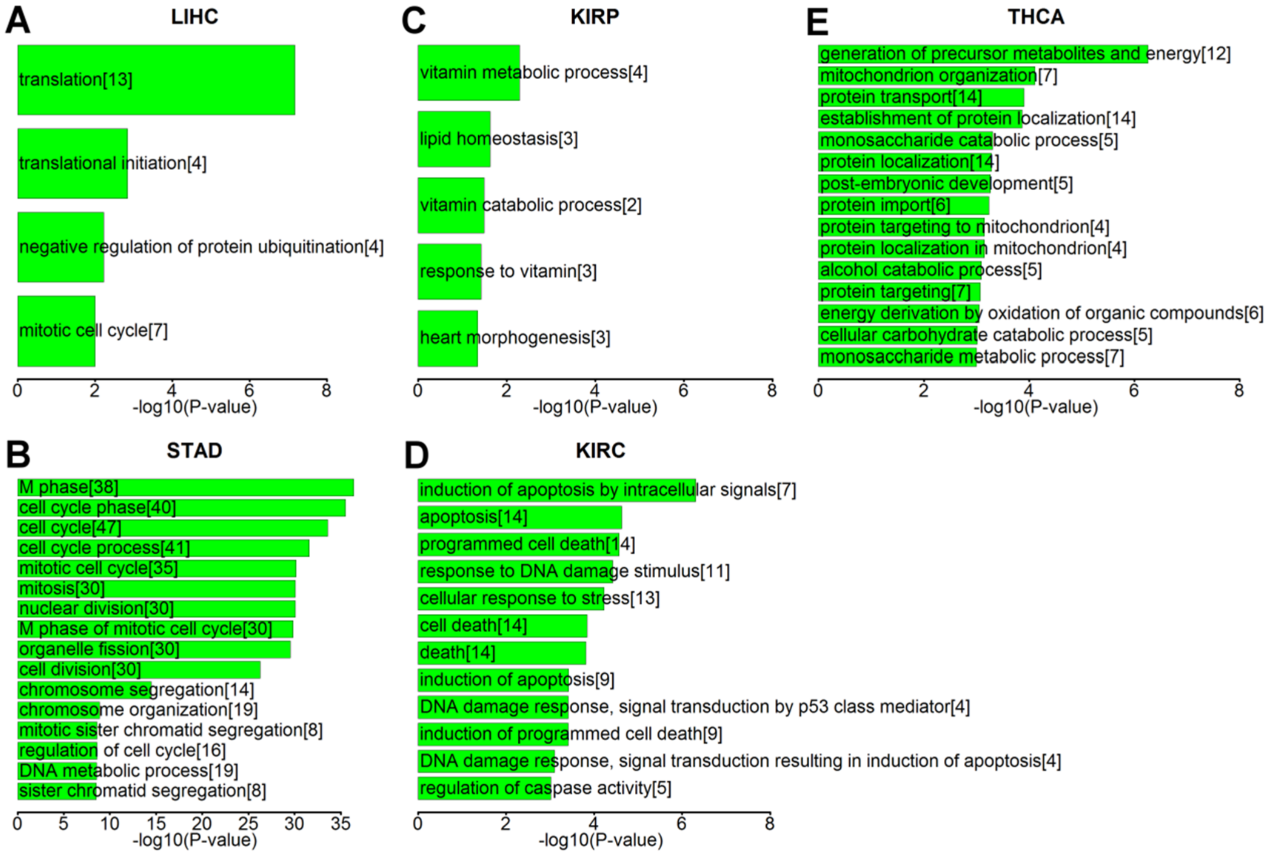


**Supplementary Figure S1: Enriched biological processes of the significantly associated genes. A.** Liver cancers. Function enrichment analysis was conducted on the top 100 genes with significant association between gene expression and A→G mutation rate. The green bars indicated the negative log10 transformed p-values for enriched biological processes. The names of biological processes were displayed on the corresponding bar, in addition to the gene number in square brackets. **B.** Stomach cancers. **C.** Renal papillary cell carcinoma. **D.** Renal clear cell carcinoma. **E.** Thyroid cancers.

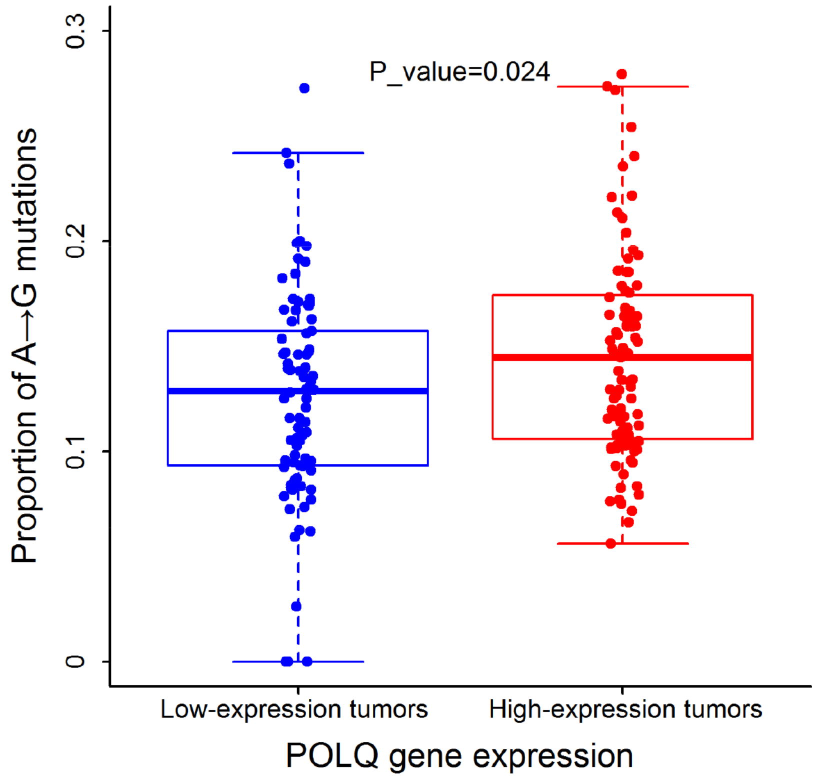


**Supplementary Figure S2: Higher proportion of A→G mutations in stomach cancers with high-expression POLQ.** Each data point represents one tumor sample. The P value for proportion difference was estimated by Mann-Whitney U test.

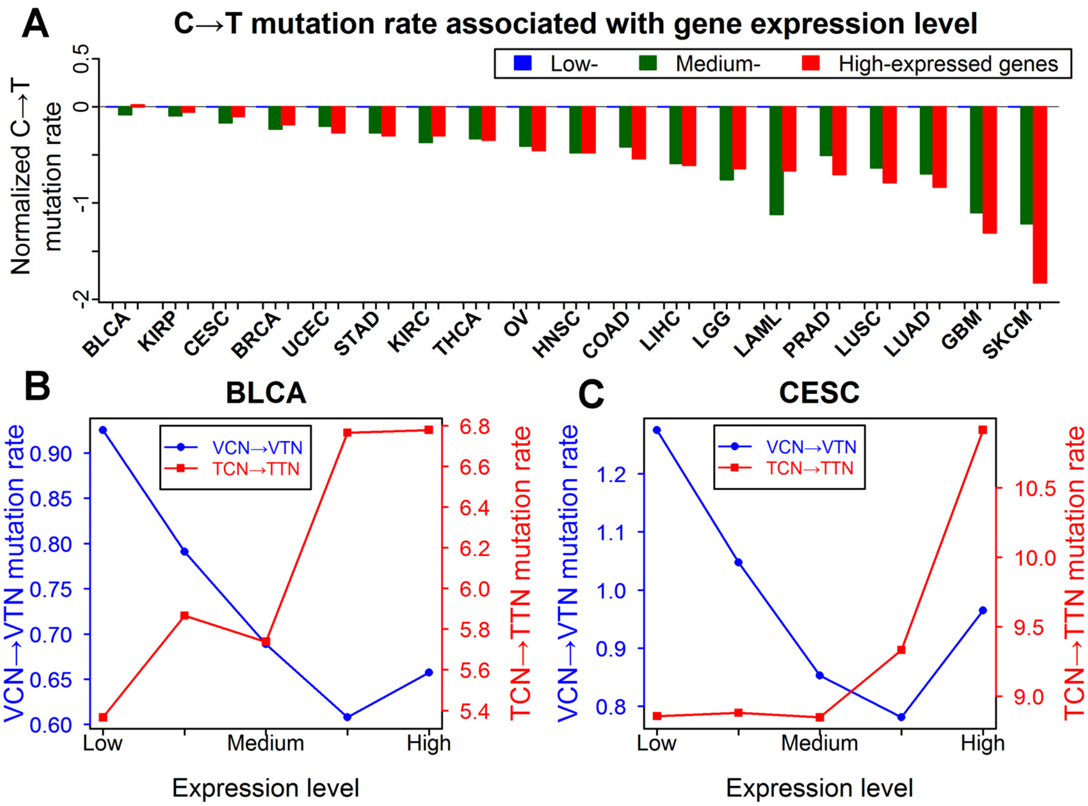


**Supplementary Figure S3: APOBEC mutagenesis associated with expression levels. A.** Higher C→T mutation rates in high-expressed genes of bladder and cervical cancers. Genes were categorized into three equal-size groups (low-, medium- and high-expressed) based on the rank of expression levels for each tumor type. The C→T mutation rates of each gene group were represented by the median value. Finally, the median rates of all gene groups were all normalized by dividing with the median rate of low-expressed genes. **B.** APOBEC mutation signatures associated with expression levels of bladder cancers. C→T mutations were divided into APOBEC signatures (TCN→TTN) and non-APOBEC signatures (VCN→VTN). V represented not-T base and N represented any base. Genes were categorized into five equal-size groups based on the rank of expression levels. The mutation rates of each gene group were represented by the median value. Blue line and red line indicated mutation rate of non-APOBEC signatures and APOBEC signatures respectively. **C.** APOBEC mutation signatures associated with expression levels of cervical cancers.

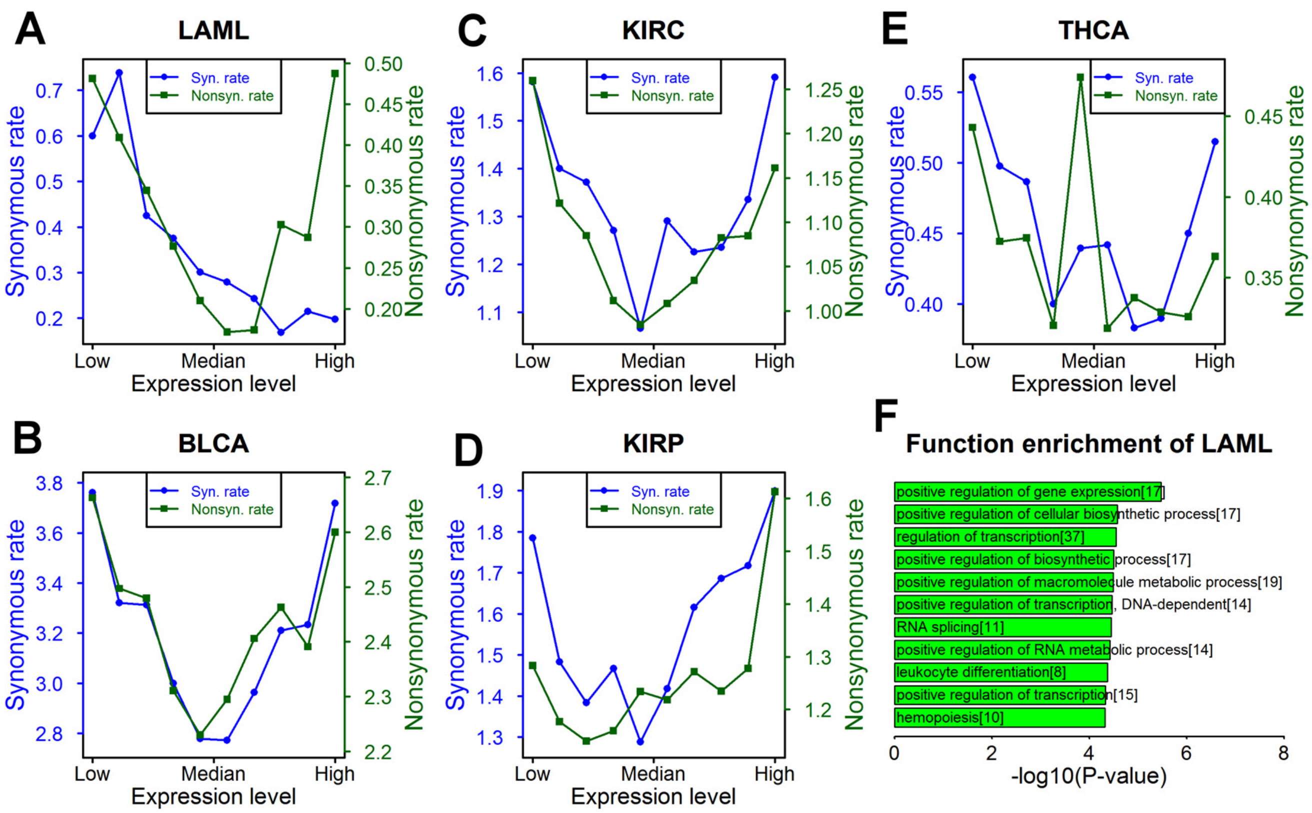


**Supplementary Figure S4: Synonymous and non-synonymous substitution rate associated with expression levels. A.** Synonymous and non-synonymous substitution rate in acute myeloid leukemia. Point mutations were divided into synonymous and non-synonymous substitutions. Genes were categorized into ten equal-size groups based on the rank of expression levels. The substitution rates of each gene group were represented by the median value. Blue line and green line indicated synonymous and non-synonymous substitution rate respectively. **B.** Bladder cancers. **C.** Renal clear cell carcinoma. **D.** Renal papillary cell carcinoma. **E.** Thyroid cancers. **F.** Enriched biological processes of the high-expressed genes with non-synonymous substitutions in acute myeloid leukemia. Function enrichment analysis was conducted on the high-expressed genes with non-synonymous substitutions. The denotation was the same as Supplementary Figure S1A.

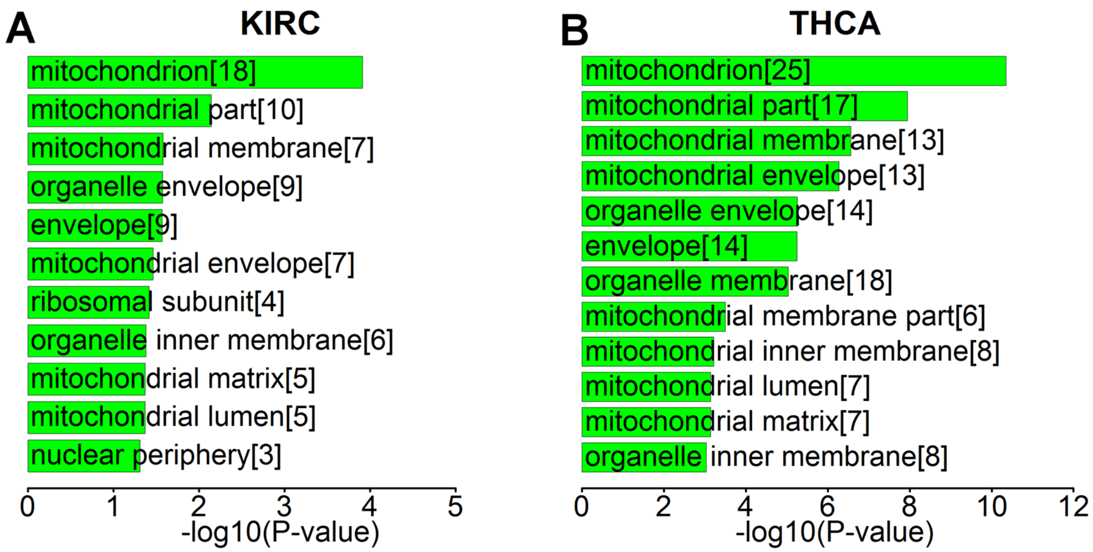


**Supplementary Figure S5: Enriched cellular components of the significantly associated genes. A.** Renal clear cell carcinoma. **B.** Thyroid cancers. Function enrichment analysis was conducted on the top 100 genes with significant association between gene expression and A→G mutation rate. The denotation was the same as Supplementary Figure S1A.

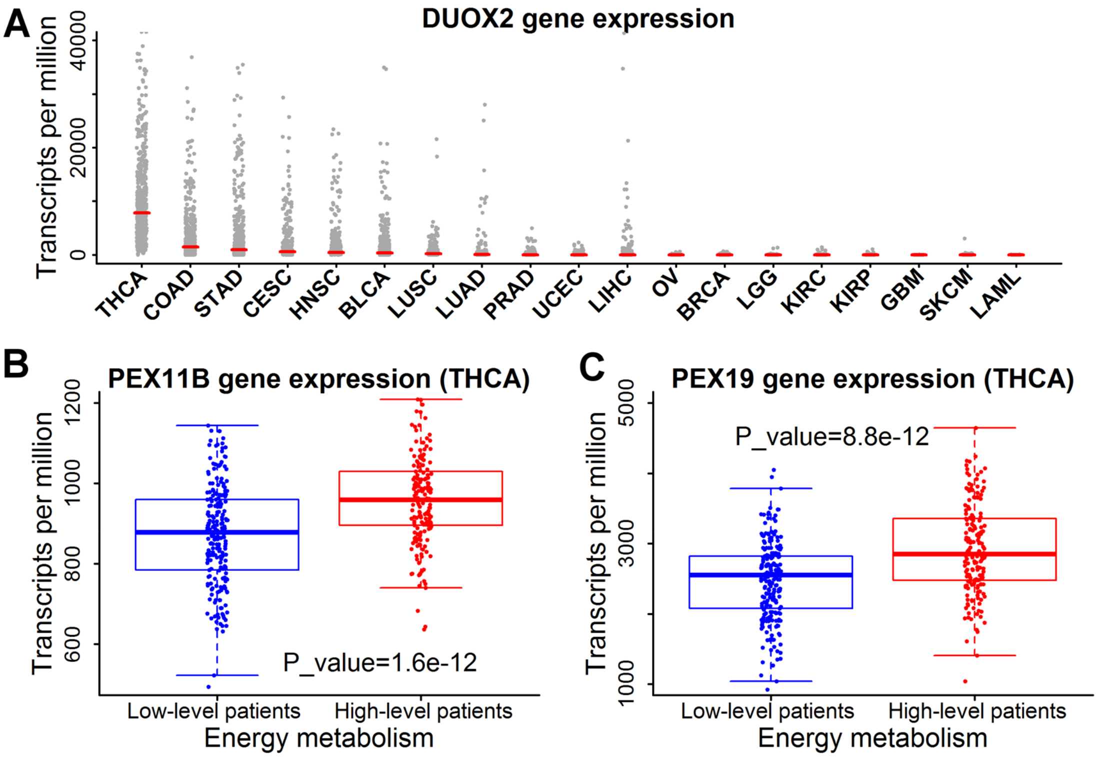


**Supplementary Figure S6: Oxidative stress associated with energy metabolism in thyroid cancers. A.** Expression levels of DUOX2 gene across 19 tumor types. Each data point represents one tumor sample. Red horizontal lines indicated median fraction of each dataset. **B.** Higher expression of PEX11B gene in thyroid cancers with high-level energy metabolism. Each data point represents one tumor sample. The P value for expression difference was estimated by Mann-Whitney U test. **C.** Higher expression of PEX19 gene in thyroid cancers with high-level energy metabolism.

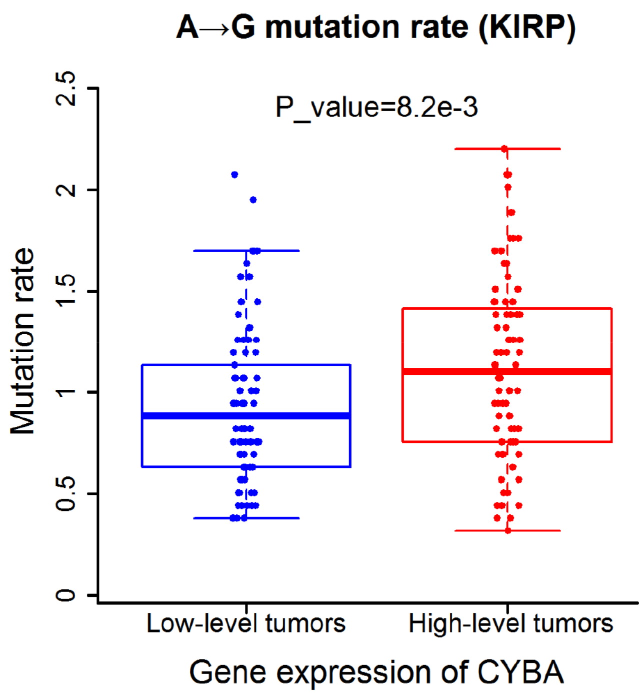


**Supplementary Figure S7: Higher A→G mutations rate in renal papillary cell carcinoma with high-expression CYBA.** Each data point represents one tumor sample. The P value for proportion difference was estimated by Mann-Whitney U test.

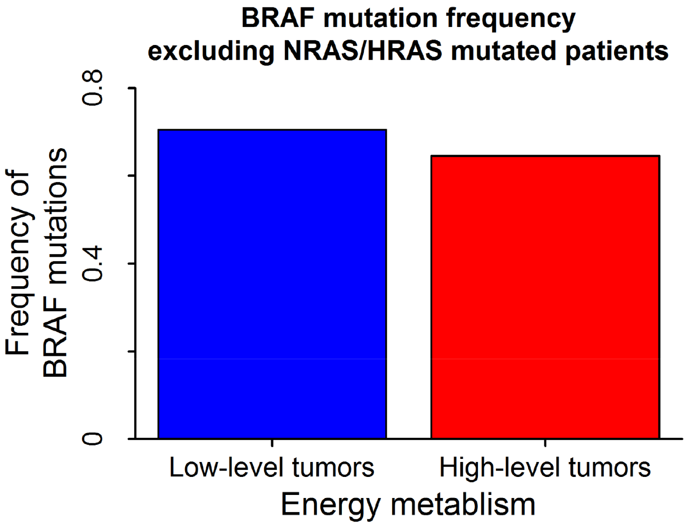


**Supplementary Figure S8: BRAF mutations associated with energy metabolism in thyroid cancers without RAS mutation.** Thyroid cancers with RAS mutations were excluded for the subsequent analyses. Blue and red bars indicated the mutation frequency of driver genes in tumors with low-level and high-level energy metabolism respectively. No significant difference of mutation frequency was observed (P= 0.246 by Fisher's exact test).

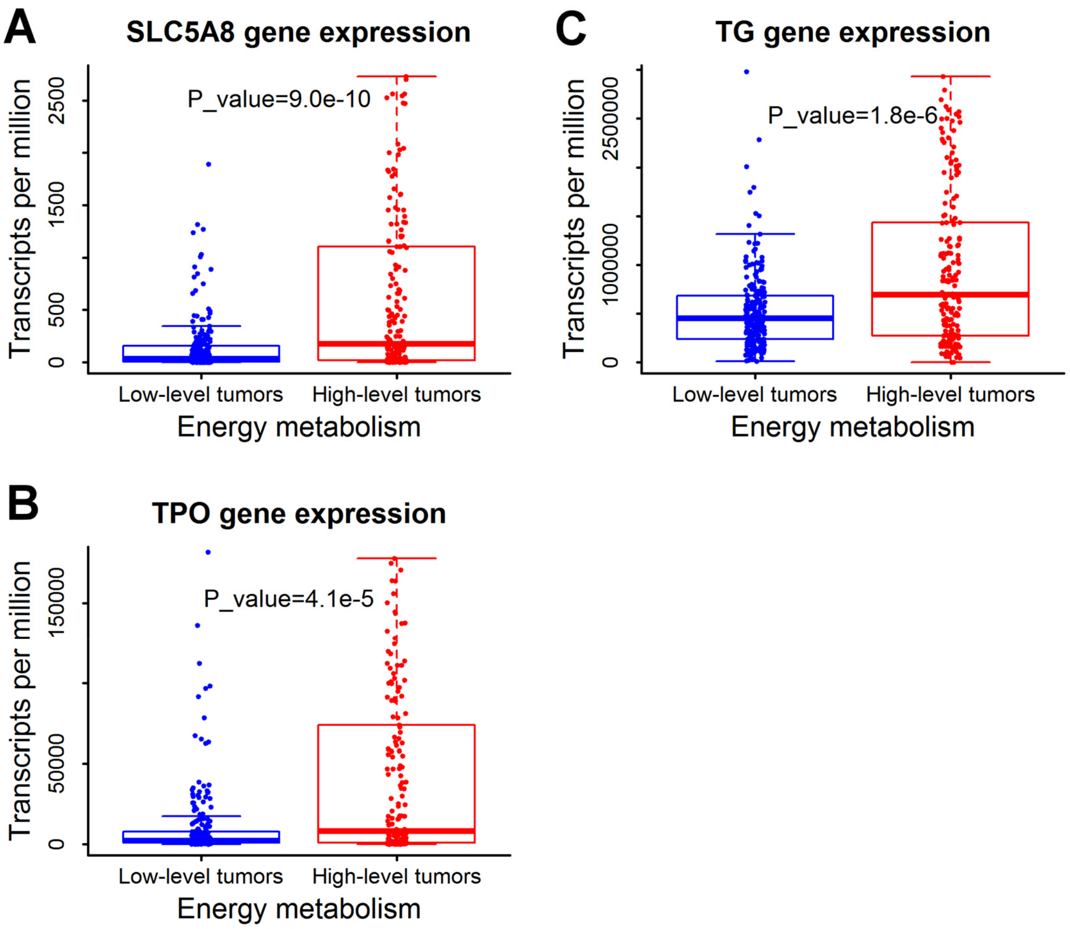


**Supplementary Figure S9: Strong association between iodine metabolism and energy metabolism.** Expression profiles were examined for three genes responsible for iodine metabolism: SLC5A8, TPO and TP genes. **A.** Higher expression of SLC5A8 gene in thyroid cancers with high-level energy metabolism. Each data point represents one tumor sample. The P value for expression difference was estimated by Mann-Whitney U test. **B.** Higher expression of TPO gene in thyroid cancers with high-level energy metabolism. **C.** Higher expression of TG gene in thyroid cancers with high-level energy metabolism.
